# 血液重症监护病房伴脓毒症血液病患者的临床特征及预后分析

**DOI:** 10.3760/cma.j.cn121090-20241204-00532

**Published:** 2025-01

**Authors:** 海涛 李, 冬雪 卢, 丹丹 李, 东阳 张, 金月 付, 倩 张, 圣瑾 范

**Affiliations:** 哈尔滨医科大学附属第一医院，黑龙江血液肿瘤研究所，血液肿瘤重症监护病房，哈尔滨 150001 The First Affiliated Hospital of Harbin Medical University, Heilongjiang Institute of Hematology & Oncology, Harbin 150001, China

**Keywords:** 粒细胞缺乏, 脓毒症, 血液病, Neutropenia, Sepsis, Hematological disease

## Abstract

**目的:**

分析血液重症监护病房（HCU）伴中性粒细胞缺乏（粒缺）脓毒症血液病患者的临床特征和预后。

**方法:**

对2017年10月至2024年10月期间哈尔滨医科大学附属第一医院HCU收治的伴脓毒症血液病患者进行回顾性分析。

**结果:**

共245例伴脓毒症血液病患者纳入研究，其中88例伴中性粒细胞缺乏（粒缺组），157例不伴中性粒细胞缺乏（非粒缺组）。急性白血病在粒缺组更为常见（55.68％，49/88）。在转入HCU时，与非粒缺组相比，粒缺组生命体征不稳定，全血细胞计数较低，炎性指标和SOFA评分较高，脓毒性休克较多见［53.40％（47/88）对36.94％（58/157），*P*<0.05］，肌酐（120.00 µmol/L对77.10 µmol/L，*P*<0.01）、总胆红素（24.70 µmol/L对17.90 µmol/L，*P*<0.01）和脑钠肽水平（567.90 ng/L对134.50 ng/L，*P*<0.01）较高。在HCU治疗期间，与非粒缺组相比，粒缺组死亡率较高［46.59％（41/88）对32.48％（51/157），*P*<0.05］，脓毒性休克是主要死亡原因（70.73％，29/41）；粒缺组革兰阴性菌［55.68％（49/88）对36.30％（57/157），*P*<0.01］和真菌［14.77％（13/88）对6.36％（10/157），*P*<0.05］较多见，肺部感染较少（*P*<0.01）。Kaplan-Meier生存曲线显示，粒缺组确诊脓毒症后28天的总生存率显著低于非粒缺组［（53.9±5.3）％对（67.7±3.7）％，*P*＝0.031］。

**结论:**

与非粒缺脓毒症患者相比，HCU中伴粒缺脓毒症血液病患者病情较重，更容易发生器官功能障碍和脓毒性休克，死亡率更高。

## Abstract (en)

**Objective:**

To identify the clinical characteristics and prognosis of patients with hematological disease and neutropenic sepsis in the hematological intensive care unit (HCU).

**Methods:**

A retrospective analysis was conducted on patients with hematological disease and sepsis who admitted to HCU, the First Affiliated Hospital of Harbin Medical University from October 2017 to October 2024, to examine the primary therapeutic options, prognosis, cause of death, and infectious features of sepsis.

**Results:**

A total of 245 septic patients were included in the study, comprising 88 cases in the neutropenic sepsis group（neutropenic group）and 157 cases in the non-neutropenic sepsis group（non-neutropenic group）. Acute leukemia was more prevalent in the neutropenic group［55.68％（49/88）］. At the time of admission to the HCU, the neutropenic group exhibited unstable vital signs, lower blood cell counts, higher inflammatory markers, elevated Sequential Organ Failure Assessment（SOFA）scores, increased creatinine levels（120.00 µmol/L *vs* 77.10 µmol/L, *P*<0.01）, higher total bilirubin levels（24.70 µmol/L *vs* 17.90 µmol/L, *P*<0.01）, and significantly elevated B-type natriuretic peptide levels（567.90 ng/L *vs* 134.50 ng/L, *P*<0.01) compared with the non-neutropenic group. Furthermore, septic shock was more common in the neutropenic group［53.40％（47/88）*vs* 36.94％（58/157）, *P*<0.05］. The mortality rate was also higher in the neutropenic group［46.59％（41/88）］compared with the non-neutropenic group［32.48％（51/157）］（*P*<0.05）, with septic shock accounting for the majority of deaths［70.73％（29/41）］. Infections caused by gram-negative bacteria［55.68％（49/88）vs 36.30％（57/157）, *P*<0.01］and fungi［14.77％（13/88）*vs* 6.36％（10/157）, *P*<0.05］were more common in the neutropenic group. However, lung infections were significantly less frequent in the neutropenic group（*P*<0.01）. Kaplan-Meier survival analysis revealed a substantially worse 28-day overall survival rate for the neutropenic group compared with the non-neutropenic group（*P*<0.05）.

**Conclusion:**

Patients with hematological diseases and neutropenic sepsis presented with more severe clinical conditions, a higher likelihood of organ failure and septic shock, and significantly increased mortality compared with patients with non-neutropenic sepsis.

脓毒症是宿主对感染反应失调所引发的比普通感染更复杂的病理生理状态，可导致危及生命的器官功能衰竭[1]。中性粒细胞是先天免疫系统的重要成员，参与防御病原体入侵和炎症反应的发展[2]。中性粒细胞缺乏（粒缺）患者常见于血液病，如恶性血液病化疗后等；因其免疫功能下降，病原体的感染风险高，粒缺患者感染的初期，炎症相关症状和体征隐秘[3]，易被忽视而迅速进展为脓毒症或脓毒性休克，需及时转入血液重症监护病房（HCU）治疗。

2018年传染病工作组（AGIHO）和德国重症监护工作组（iCHOP）发布了癌症患者中性粒细胞减少脓毒症处理指南[4]。该指南指出，虽然粒缺脓毒症与非粒缺脓毒症的病理生理并无显著差异，但粒缺脓毒症的诊断和治疗仍有特殊之处。另有少量研究表明，相对于非粒缺脓毒症患者而言，粒缺脓毒症患者更容易发生脓毒性休克（43.6％对22.9％，*P*<0.01），死亡率更高（42.2％对26.3％，*P*<0.01）[5]。粒缺患者感染初期，虽然炎症反应不明显，但当进展为脓毒症时，可表现出比非粒缺脓毒症更明显的炎症反应状态，并且这种高炎症反应状态与重要脏器损伤的发生和高死亡率密切相关[6]。

鉴于粒缺脓毒症的上述特点，本研究回顾性分析了HCU收治的伴脓毒症血液病患者的临床特征并进行预后分析，以了解粒缺和非粒缺脓毒症血液病患者的差异，便于血液科医生和ICU医生更及时地发现和管理伴脓毒症血液病患者，提高患者存活率。

## 病例与方法

一、病例

本研究为单中心回顾性研究，纳入2017年10月至2024年10月期间哈尔滨医科大学附属第一医院HCU收治的伴脓毒症血液病患者。本研究获得哈尔滨医科大学附属第一医院伦理委员会批准。纳入标准：①年龄≥18岁；②符合脓毒症诊断标准；③原发基础疾病为血液病。排除标准：①新型冠状病毒感染；②既往慢性肾功能不全；③HCU治疗过程中放弃治疗；④转入其他科室治疗。

根据患者在HCU中是否合并粒缺，将符合条件的患者分为非粒缺组和粒缺组。

收集患者以下临床资料：①性别、年龄、基础疾病；②转入HCU时的生命体征，序贯器官衰竭评分（SOFA评分），实验室检查等；③HCU治疗期间感染特征（感染部位、病原体类型、确认病原体的标本等）；④新发不良事件和主要死亡原因；⑤HCU的治疗措施［经鼻高流量氧疗、机械通气、连续肾脏替代疗法（CCRT）、血管活性药物等］。

二、相关定义

根据脓毒症-3.0进行脓毒症诊断，即感染基础上，伴SOFA评分急性改变≥2分[1]。

根据2018年AGIHO和iCHOP发布的癌症患者中性粒细胞减少脓毒症处理指南[4]，粒缺脓毒症的诊断需满足以下条件：①患者外周血中性粒细胞绝对计数（ANC）<0.5×10^9^/L，或ANC<1.0×10^9^/L具预计2 d内下降至<0.5×10^9^/L；②合并或不合并发热；③SOFA评分急性改变≥2分。

如果患者符合脓毒症或粒缺脓毒症的标准，出现持续性低血压，在充分容量复苏后仍需血管活性药来维持平均动脉压（MAP）≥65 mmHg，并且血乳酸浓度>2 mmol/L，即可诊断为脓毒性休克或粒缺脓毒性休克[4]。

三、脓毒症患者的治疗方案

转入HCU的脓毒症患者均接受标准的重症监护治疗及护理方案。呼吸衰竭患者给予经鼻高流量氧疗或机械通气治疗；确诊脓毒症后，给予抗感染、液体复苏、联合血管活性药物等治疗；对于血肌酐（Cr）显著升高、尿量减少和容量超负荷的急性肾功能衰竭患者，给予CRRT。

治疗过程中PLT≤10×10^9^/L时常规输注血小板；纤维蛋白原（FIB）≤1 g/L时输注纤维蛋白原，凝血酶原时间（PT）延长≥3 s或活化部分凝血活酶时间（APPT）延长≥10 s时，予以新鲜冰冻血浆400 ml输注；HGB<70 g/L时，予以浓缩红细胞输注。

四、统计学处理

非正态分布的计量资料用“中位数（四分位间距）”表示，组间比较采用Mann-Whitney *U*非参数检验；计数资料组间比较采用卡方检验；生存分析采用Kaplan-Meier生存曲线法，两组之间生存曲线的比较采用Log-rank检验；使用SPSS 25.0软件进行统计学分析，*P*<0.05被认为差异有统计学意义；使用Graphpad Prism 8.0软件进行绘图。

## 结果

一、伴脓毒症血液病患者的临床特征

245例患者被纳入本研究，其中非粒缺组157例，粒缺组88例。性别、年龄和既往慢性基础疾病在粒缺和非粒缺组间差异无统计学意义；病种分布在粒缺和非粒缺组间差异有统计学意义（*P*<0.01），其中急性白血病占粒缺组55.68％（49/88）。

转入HCU当日，与非粒缺组相比，粒缺组生命体征相对不稳定，即更高的体温（38.05 °C对37.20 °C，*P*<0.01），更快的呼吸频率（27.50次/分对25.00次/分，*P*<0.05）和心率（121次/分对110次/分，*P*<0.01），以及更低的平均血压（65 mmHg对70 mmHg，*P*<0.01）；同时，粒缺组有更低的WBC（0.29×10^9^/L对9.31×10^9^/L，*P*<0.01）、HGB（70.31 g/L对79.00 g/L，*P*<0.05）和PLT（11×10^9^/L对36×10^9^/L，*P*<0.01）；粒缺组C反应蛋白（CRP）、白介素6（IL-6）和降钙素原（PCT）升高更明显（CRP：180.50 mg/L对92.60 mg/L，*P*<0.01；IL-6：1097.15 ng/L对43.67 ng/L，*P*<0.01；PCT：16.75 µg/L对2.68 µg/L，*P*<0.01）；与非粒缺组相比，粒缺组脓毒性休克较常见［53.40％（47/88）对36.94％（58/157），*P*<0.05］，SOFA评分较高（11分对9分，*P*<0.01），Cr、总胆红素（TBIL）和脑钠肽（BNP）水平也较高（Cr：120.00 µmol/L对77.10 µmol/L，*P*<0.01；TBIL：24.70 µmol/L对17.90 µmol/L，*P*<0.05；BNP：567.9 ng/L对134.5 ng/L，*P*<0.01），详见表1。

**表1:** 245例血液重症监护病房伴脓毒症血液病患者一般资料

基本特征	非粒缺组（157例）	粒缺组（88例）	统计量	*P*值
性别［例（％）］				
男	100（40.82）	48（19.59）	1.974	0.160
女	57（23.27）	40（16.33）		
年龄［岁，*M*（*IQR*）］	61（46.50，69.00）	59（48.50，66.00）	0.754	0.451
基础血液病［例（％）］				
急性白血病	51（20.82）	49（20.00）	26.000	<0.001
淋巴瘤	21（8.57）	13（5.31）		
骨髓增生异常综合征	13（5.31）	9（3.67）		
再生障碍性贫血	5（2.04）	7（2.86）		
多发性骨髓瘤	15（6.12）	0		
其他	52（21.22）	10（4.08）		
慢性基础疾病［例（％）］				
糖尿病	21（13.37）	11（12.50）	0.038	0.845
冠心病	14（8.91）	6（6.81）	0.331	0.565
高血压	33（21.01）	14（15.90）	0.950	0.330
慢性肝病	10（6.36）	4（4.54）	0.348	0.555
入HCU时生命体征［*M*（*IQR*）］				
体温（°C）	37.20（36.50，38.27）	38.05（37.00，39.57）	3.014	0.003
呼吸频率（次/分）	25（20，30）	27（22，35）	2.391	0.017
心率（次/分）	110（93，125）	121（107，139）	6.571	<0.001
平均血压（mmHg）	70（61，90）	65（58，74）	6.324	<0.001
脓毒性休克［例（％）］	58（36.94）	47（53.40）	6.244	0.012
SOFA评分［*M*（*IQR*）］	9（8，12）	11（9，13）	5.698	<0.001
实验室检查［*M*（*IQR*）］				
WBC（×10^9^/L）	9.31（4.00，21.45）	0.29（0.12，0.52）	12.471	<0.001
HGB（g/L）	79.00（62.00，91.50）	70.31（58.56，83.00）	2.638	0.008
PLT（×10^9^/L）	36（17.00，90.72）	11（5.10，23.00）	6.792	<0.001
CRP（mg/L）	92.60（37.60，204.00）	180.50（77.17，280.75）	3.157	0.002
IL-6（ng/L）	43.67（21.10，238.70）	1097.15（241.50，4221.43）	3.439	<0.001
PCT（µg/L）	2.68（0.68，16.78）	16.75（2.60，62.67）	3.882	<0.001
Cr（µmol/L）	77.10（54.50，117.00）	120.00（62.00，275.00）	3.574	<0.001
TBIL（µmol/L）	17.90（10.90，39.25）	24.70（14.19，47.18）	2.415	0.016
ALT（U/L）	32.20（15.00，58.25）	28.00（15.50，43.20）	0.550	0.582
PT（s）	15.05（13.17，17.82）	15.30（13.40，16.85）	0.143	0.886
D-二聚体（mg/L）	3.97（1.76，12.19）	3.80（1.43，7.12）	1.302	0.193
BNP（ng/L）	134.50（75.70，634.40）	567.90（122.45，1882.90）	2.662	0.008
动脉血乳酸浓度（mmol/L）	4.30（2.35，10.55）	5.35（2.65，9.55）	0.203	0.839
HCU住院时间［d，*M*（*IQR*）］	4（2，6）	3（2，6）	1.735	0.083

**注** BNP：脑钠肽；Cr：肌酐；CRP：C反应蛋白；CRRT：连续肾脏替代疗法；IL-6：白介素6；PCT：降钙素原；PT：凝血酶原时间；TBIL：总胆红素

二、伴脓毒症血液病患者的感染特征

本研究中，最常见的感染部位为肺感染（59.59％）。与非粒缺组相比，粒缺组肺感染相对较少（47.72％对66.24％，*P*<0.01），其他感染部位分布差异无统计学意义。感染部位的数量在两组间差异无统计学意义。与非粒缺组相比，粒缺组革兰阴性菌（55.68％对36.3％，*P*<0.01）和真菌（14.77％对6.36％，*P*<0.05）感染更多见，详见表2。

**表2:** 血液重症监护病房脓毒症患者的感染特征［例（％）］

变量	非粒缺脓毒症（157例）	粒缺脓毒症（88例）	*χ*^2^值	*P*值
主要感染部位				
肺部	104（66.24）	42（47.72）	8.028	0.005
血流	50（31.84）	37（42.04）	2.561	0.110
肠道	10（6.36）	10（11.36）	1.876	0.171
皮肤软组织	10（6.36）	9（10.22）	1.173	0.279
感染部位数量				
1个	108（44.08）	51（20.82）	3.065	0.216
≥2个	35（14.29）	25（10.20）		
不明	14（5.71）	12（4.90）		
病原体				
革兰阴性菌	57（36.30）	49（55.68）	8.625	0.003
革兰阳性菌	28（17.83）	16（18.18）	0.005	0.946
真菌	10（6.36）	13（14.77）	4.681	0.030
病毒	9（5.73）	1（1.13）	3.030	0.073
确认病原体的标本				
血液	45（28.66）	26（29.54）	0.021	0.884
痰或肺泡灌洗液	31（19.74）	15（17.04）	0.270	0.604
肛拭子	15（9.55）	7（7.95）	0.176	0.433
脓液	1（0.63）	4（4.54）	4.292	0.057
胸腔引流液	3（1.91）	0	1.702	0.192

**注** HCU：血液重症监护病房

三、伴脓毒症血液病患者的治疗

HCU治疗期间，粒缺组接受血管活性药物治疗的患者占比高于非粒缺组［53.40％（47/88）对36.94％（58/157），*P*＝0.017］，接受抗生素治疗、经鼻高流量氧疗、机械通气及CRRT的患者占比两组间差异均无统计学意义，详见表3。

**表3:** 血液重症监护病房脓毒症患者的治疗措施［例（％）］

变量	非粒缺组（157例）	粒缺组（88例）	*χ*^2^值	*P*值
抗生素治疗				
单药	23（9.39）	7（2.86）	2.343	0.089
联合	134（54.69）	81（33.06）		
血管活性药物	58（36.94）	47（53.4）	6.244	0.017
机械通气	53（21.63）	34（13.88）	0.584	0.265
经鼻高流量氧疗	20（8.16）	13（5.31）	0.199	0.396
CRRT	28（11.43）	8（3.27）	3.439	0.064

**注** CRRT：连续肾脏替代疗法

四、伴脓毒症血液病患者的死亡原因

本研究纳入的245例伴脓毒症血液病患者中，HCU中共死亡92例（37.55％）。与非粒缺组相比，粒缺组患者死亡率更高［46.59％（41/88）对32.48％（51/157），*P*<0.05］。所有死亡患者中，90例多于HCU治疗期间死亡，另有2例转入普通病房后死亡。粒缺组、非粒缺组确诊脓毒症后28 d的总生存率分别为（53.9±5.3）％、（67.7±3.7）％（*P*＝0.031）。

两组间死亡原因差异具有统计学意义（*P*<0.01）。脓毒性休克是最主要的死亡病因（55.43％），其次依次为肺感染继发呼吸衰竭（23.91％）、肺出血继发呼吸衰竭（7.61％）、脑出血（6.52％）、心源性休克（4.35％）和颅内感染（2.17％）。其中粒缺组因脓毒性休克死亡的患者占多数（29/41，70.73％），见表4。

**表4:** 血液重症监护病房中伴脓毒症血液病患者的主要死亡原因［例（％）］

变量	非粒缺组（157例）	粒缺组（88例）	*χ*^2^值	*P*值
死亡人数	51（32.48）	41（46.59）	4.786	0.029
死亡原因				
脓毒性休克	22（23.91）	29（31.52）	8.000	0.005
肺感染并发呼吸衰竭	18（19.57）	4（4.35）		
肺出血并发呼吸衰竭	2（2.17）	5（5.43）		
脑出血	3（3.26）	3（3.26）		
心源性休克	4（4.35）	0		
颅内感染	2（2.17）	0		

## 讨论

粒缺患者是一组特殊的疾病人群，由于免疫功能低下，易发生细菌或真菌等病原体感染，且易迅速进展为脓毒症。在HCU中，粒缺脓毒症患者最常见于急性白血病化疗后，如果患者粒细胞恢复则可能获得较高的存活率。本研究分析了HCU中血液病粒缺脓毒症患者的临床特征和死亡原因，结果提示，相对于非粒缺脓毒症，粒缺脓毒症患者病情更复杂，预后较差，脓毒性休克是其主要的死亡病因。

韩国脓毒症联盟的一项多中心队列研究分析了2019年至2020年期间成人脓毒症患者的数据，共收集了2 074例脓毒症患者（粒缺组1 856例，非粒缺组218例），粒缺组住院死亡率较高（42.2％对26.3％，*P*<0.01），脓毒性休克发生率较高（43.6％对22.9％，*P*<0.01），SOFA评分较高（7分对5分，*P*<0.01）[5]。提示伴粒缺脓毒症具有特殊的临床特征，但该研究并不限于血液病患者。

本研究报道了本中心HCU中伴粒缺脓毒症血液病患者的临床特征，结果与上述研究基本一致。与非粒缺脓毒症患者相比，粒缺脓毒症患者在转入HCU时的生命体征相对不稳定、脓毒性休克更多见，因此在HCU治疗期间，更多患者接受血管活性药物治疗，并且死亡风险更高。其原因在于粒缺患者感染时，炎症相关症状和体征通常不明显，或仅以发热为唯一征象，感染部位不明显或较难发现，易被普通病房临床医生忽视[7]。而粒缺患者感染发展迅速，当转入HCU时，或已进展为严重脓毒症或脓毒性休克。脓毒性休克是脓毒症进展最严重的阶段，死亡风险高，这是导致HCU中粒缺脓毒症患者高死亡率的重要原因之一。

相对于非粒缺脓毒症，粒缺脓毒症的高炎症反应导致的脏器损伤可能更明显。粒缺脓毒症患者虽然粒细胞缺乏，但淋巴细胞或巨噬细胞等免疫细胞亦可被激活并释放细胞因子和炎症介质，参与脏器损伤。一项单中心前瞻性研究[6]纳入了797例脓毒症患者（粒缺患者103例），粒缺组的炎症因子水平，包括血浆IL-6（457 ng/L对249 ng/L，*P*<0.05）、IL-8（581 ng/L对94 ng/L，*P*<0.01）和粒细胞集落刺激因子（G-CSF）（3624 ng/L对99 ng/L，*P*<0.01）水平均高于非粒缺组，且上述炎症因子水平与高病死率和急性肾损伤的发生密切相关。该研究分析其原因，即典型的抗原提呈细胞吞噬衰老的中性粒细胞，抑制粒细胞生成，但由于中性粒细胞缺乏，规避了这种负反馈机制，从而导致G-CSF和IL-6水平持续升高以刺激中性粒细胞产生IL-8以趋化中性粒细胞。

本研究结果显示，当患者转入HCU时，与非粒缺组相比，粒缺脓毒症患者表现更明显的高炎症反应状态，即体温更高、呼吸频率和心率更快，CRP、IL-6和PCT水平更高，还伴更严重的脏器损伤（肌酐、胆红素水平、BNP和SOFA评分均显著增高）。另外，在HCU治疗过程中，与非粒缺组相比，粒缺组发生肾损伤较多。由于促炎因子或高炎症反应状态和器官功能障碍的发生被认为是死亡的独立危险因素[8]–[9]，因此，伴粒缺脓毒症患者发生器官功能障碍和脓毒性休克可能更多归因于严重的免疫失调，这也是导致伴粒缺脓毒症患者易发生肾损伤和高死亡率的重要原因之一。

病原流行病学调查结果显示，我国粒缺伴发热患者的致病微生物分布以革兰阴性菌为主，占全部细菌总数的54.0％；在血流感染患者中，革兰阴性菌比例可高达81.3％[10]–[12]。在本研究中，伴粒缺脓毒症血液病患者的病原特征与我国粒缺伴发热患者的病原特征基本相符，脓毒症患者的致病菌以革兰阴性菌为主。与非粒缺组相比，粒缺组革兰阴性菌和真菌更多见。本研究中，脓毒症的患者主要感染部位为肺部，但粒缺组肺部感染发生相对较少。其原因在于，中性粒细胞是抵抗病原体感染的重要防御系统之一，由于中性粒细胞缺乏，患者不仅感染风险增加，而且因免疫系统抑制，导致感染灶不易局限于特定器官，更容易通过血液或其他途径传播。

本研究存在一定的不足：①回顾性研究，可能存在选择偏倚；②样本量较少，需扩大样本量或多中心研究进一步证实本研究结论；③我院炎症系列指标的检测开展较晚，包括肿瘤坏死因子α、IL-8和IL-10等炎症指标缺失值较多，仅评估了脓毒症患者IL-6、CRP和PCT水平。

综上所述，HCU粒缺与非粒缺脓毒症血液病患者临床特点不同，伴粒缺患者病情更重，死亡率更高。认识粒缺脓毒症特点对早期诊断和治疗有重要价值。
